# Additional value of EUS in oesophageal cancer patients staged N0 on PET/CT: validation of a prognostic model

**DOI:** 10.1007/s00464-018-6259-0

**Published:** 2018-06-04

**Authors:** K. G. Foley, P. Fielding, W. G. Lewis, S. A. Roberts

**Affiliations:** 1 Division of Cancer & Genetics, School of Medicine, Cardiff University, Cardiff, CF14 4XN UK; 2 Wales Research and Diagnostic Positron Emission Tomography Imaging Centre (PETIC), Cardiff, UK; 3 Department of Surgery, University Hospital of Wales, Cardiff, UK; 4 Department of Radiology, University Hospital of Wales, Cardiff, UK

**Keywords:** Oesophageal cancer, Endoscopic ultrasound, Positron-emission tomography, Prognosis, Survival

## Abstract

**Background:**

Lymph node metastases are a major prognostic indicator in oesophageal cancer. Radiological staging largely influences treatment decisions and is becoming more reliant on PET and CT. However, the sensitivity of these modalities is suboptimal and is known to under-stage disease. The primary aim of this study was to validate a published prognostic model in oesophageal cancer patients staged N0 with PET/CT, which showed that EUS nodal status was an independent predictor of survival. The secondary aim was to assess the prognostic significance of pathological lymph node metastases in this cohort.

**Methods:**

An independent validation cohort included 139 consecutive patients from a regional upper gastrointestinal cancer network staged N0 with PET/CT between 1st January 2013 and 31st June 2015. Replicating the original study, two Cox regression models were produced: one included EUS T-stage and EUS N-stage, and one included EUS T-stage and EUS N0 versus N+. The primary outcome of the prognostic model was overall survival (OS). Kaplan–Meier analysis assessed differences in OS between pathological node-negative (pN0) and node-positive (pN+) groups. A *p* value of < 0.05 was considered statistically significant.

**Results:**

The mean OS of the validation cohort was 29.8 months (95% CI 27.1–35.2). EUS T-stage was significantly and independently associated with OS in both models (*p* = 0.011 and *p* = 0.012, respectively). EUS N-stage and EUS N0 versus N+ were not significantly associated with OS (*p* = 0.553 and *p* = 0.359, respectively). There was a significant difference in OS between pN0 and pN+ groups (*χ*^2^ 13.315, *df* 1, *p* < 0.001).

**Conclusion:**

Lymph node metastases have a significant detrimental effect on OS. This validation study did not replicate the results of the developed prognostic model but the continued benefit of EUS in patients staged N0 with PET/CT was demonstrated. EUS remains a valuable component of a multi-modality approach to oesophageal cancer staging.

In oesophageal cancer, clinical management is largely influenced by radiological lymph node staging. Accurate radiological staging is therefore vital to guide optimum treatment decisions for each patient. Patients considered to have lymph node metastases are more likely to receive neo-adjuvant therapy prior to surgery, routinely involving a combination of chemotherapy and radiotherapy [1].

Under-staging of lymph node metastases increases the likelihood of recurrence and has a negative impact upon overall survival (OS) [2]. Under-staging of disease can also impact on patient’s quality of life, potentially exposing them to high-risk surgical resection or definitive chemo-radiotherapy with toxic side-effects [3]. Currently, patients considered to be suitable for radical therapy undergo PET/CT and EUS for further detailed assessment of local and distant disease [4]. However, EUS utilisation in the UK is declining. Data from the National Oesophago-Gastric Cancer Audit (NOGCA) show 47.5% of patients had staging EUS in 2016 compared to 62% in 2013 [5].

As a result, there is an increasing reliance on cross-sectional imaging for treatment planning. The low sensitivity of PET/CT can increase the false-negative diagnostic rate for lymph node metastases, under-staging regional disease and contributing to suboptimal treatment decisions [6].

A prognostic model investigating the additional role of EUS in patients staged N0 on PET/CT was developed [7]. The results of the study showed a significant difference in OS in patients with EUS-positive nodal disease compared to EUS N0 disease. The inherently poor spatial resolution of PET was thought to affect the differentiation of peri-tumoural nodes and the detection of small lymph node metastases, compared to the superior spatial resolution of EUS [8].

The primary aim of this study was to validate the prognostic model in an independent cohort of oesophageal cancer patients staged N0 with PET/CT. The secondary aim was to assess the prognostic significance of pathological lymph node metastases in these patients.

## Materials and methods

This was a validation study of a previously published prognostic model [7]. The setting was a regional upper gastrointestinal cancer network serving a population of 1.5 million. The prognostic model was developed in patients staged with PET/CT in two centres (sites 1 and 2). Validation was conducted in an independent cohort of patients staged N0 with PET/CT in site 2 only. Scientific review by the Research Review Board was performed and institutional research approval was obtained (reference 13//DMD5769). Formal ethical approval was not required for this study.

### Development and validation cohorts

One-hundred and seventeen patients were included in the development patient cohort. Consecutive patients were staged N0 with PET/CT between 1st December 2008 and 31st May 2012. PET/CT examinations were performed across 2 sites; 47 in the first centre (site 1) and 70 in the second centre (site 2). The PET/CT protocols were previously published [7]. Patients with M1 disease (non-regional nodal disease or distant metastases) on PET/CT (*n* = 6) or those with incomplete EUS staging (*n* = 39) were excluded. All patients were staged N0 on PET/CT by a Consultant Radiologist certified in PET/CT reporting and had staging EUS completed. All staging was classified according to the TNM 7th edition [9].

The same selection criteria were applied to the validation cohort. Patients staged N0 with PET/CT between 1st January 2013 and 31st June 2015 were considered for the validation cohort (*n* = 166). Patients with distant metastases (*n* = 4) or incomplete EUS staging (*n* = 23) were excluded. Following exclusions, 139 patients were included in the validation study. All patients in the validation cohort followed the usual staging pathway and had PET/CT in site 2 using the same scanner and protocol [7]. All patients had complete EUS staging using the same technique as described in the development cohort [7]. All staging was classified according to the TNM 7th edition [9]. As in the previous study, 2 variables were recorded for each patient: EUS T-stage (T1–4a) and EUS N-stage (N0–3). A third variable was derived from the EUS N-stage: EUS N0 versus N+ (N1, N2 or N3).

### Survival data

The primary outcome of the study was OS, defined in months from the date of diagnosis. Survival data were updated in July 2016 ensuring at least 12 months of follow-up per patient. Patients were followed-up every 3 months for the first year, then every 6 months thereafter. No patients were lost to follow-up.

### Statistical analysis

Continuous data were expressed as median (range) and categorical data as frequency (%). Univariate analysis was performed for EUS T-stage, EUS N-stage, and EUS N0 versus N+, and differences between groups assessed using the log-rank test [10]. Two Cox regression models were constructed to assess the independent prognostic value of variables; model 1 included EUS T-stage and EUS N-stage and model 2 included EUS T-stage and EUS N0 versus N+ [11]. Kaplan–Meier analysis using the log-rank test assessed differences in OS between the development and validation cohorts. In addition, model discrimination was assessed using a log-rank test to evaluate OS differences between pN0 and pN+ groups in the sub-group of patients who underwent surgical resection in the validation cohort. The effect of two different PET/CT scanners in the development study was further investigated by excluding patients from site 1 and re-calculating the models. The event-per-variable (EPV) ratio ensured that the study was adequately powered, with a minimum EPV of 10 recommended, and an event defined as a death [12]. A *p* value of < 0.05 was considered statistically significant. Statistical analysis was performed with SPSS v23 (IBM, Chicago, USA). This validation study is reported according to the Transparent Reporting of a Multivariable Prediction Model for Individual Prognosis or Diagnosis (TRIPOD) guidelines [13].

## Results

### Patient cohorts

The baseline characteristics of development and validation patient cohorts are included in Table 1. There were no missing data in this study. The median age of the development and validation cohorts was 67.0 years (range 24.0–82.0) and 66.0 years (39–84), respectively. The median follow-up period was 25.0 months in the development cohort (95% CI 23.1–26.9) and 26.0 months (95% CI 22.7–29.3) in the validation cohort. Mean survival times are presented because a 50% mortality rate was not reached in either cohort. The mean OS of the development cohort was 33.1 months (95% CI 30.1–36.1) and 29.8 months (95% CI 27.1–35.2) in the validation cohort.

**Table 1:** Baseline characteristics of patients in development and validation cohorts

Frequency (%)	Development cohort (*n* = 117)	Validation cohort (*n* = 139)
Male:female	88 (75.2):29 (24.8)	108 (77.7):31 (22.3)
Tumour location		
Oesophagus	73 (62.4)	76 (54.7)
Upper	0 (0.0)	2 (2.7)
Mid	20 (27.4)	22 (28.9)
Distal	53 (72.6)	52 (68.4)
Junction	44 (37.6)	63 (45.3)
Siewert type I	5 (11.3)	25 (39.7)
Siewert type II	12 (27.3)	18 (28.6)
Siewert type III	27 (61.4)	20 (31.7)
Histology		
Adenocarcinoma	98 (83.8)	107 (77.0)
SCC	19 (16.2)	26 (18.7)
HGD	0 (0.0)	3 (2.2)
Neuro-endocrine	0 (0.0)	2 (1.4)
Undifferentiated	0 (0.0)	1 (0.7)
EUS T-stage		
T1	18 (15.4)	20 (14.4)
T2	16 (13.7)	18 (12.9)
T3	75 (64.1)	86 (61.9)
T4a	8 (6.8)	13 (9.4)
T4b	0 (0.0)	2 (1.4)
EUS N-stage		
N0	78 (66.7)	89 (64.0)
N1	23 (19.7)	42 (30.2)
N2	9 (7.6)	7 (5.1)
N3	7 (6.0)	1 (0.7)
Treatment		
Curative	105 (89.7)	116 (83.5)
NACT	40 (38.1)	44 (37.9)
dCRT	29 (27.6)	39 (33.6)
Surgery alone	32 (30.5)	19 (16.4)
NACRT	1 (0.9)	11 (9.5)
EMR	3 (2.9)	3 (2.6)
Palliative	12 (10.3)	23 (16.5)
Mortality		
Alive	84 (71.8)	85 (61.2)
Dead	33 (28.2)	54 (38.8)

### Summary of results from development cohort

Univariate analysis showed that EUS T-stage (Χ^2^ 8.321, *df* 3, *p* = 0.040), N-stage (Χ^2^ 14.879, *df* 3, *p* = 0.002), and N0 versus N+ (*χ*^2^ 11.325, *df* 1, *p* = 0.001) were significantly associated with OS. When EUS T-stage and N-stage were entered in model 1, only EUS N-stage was significantly and independently associated with duration of survival [hazard ratio (HR) 1.616–4.707, 95% CI 0.363–7.190, *p* = 0.005]. When EUS T-stage and EUS N0 versus N+ were entered in model 2, EUS N0 versus N+ was significantly and independently associated with OS (HR 3.105, 95% CI 1.543–6.247, *p* = 0.001).

### Univariate analysis in validation cohort

**Fig. 1:**
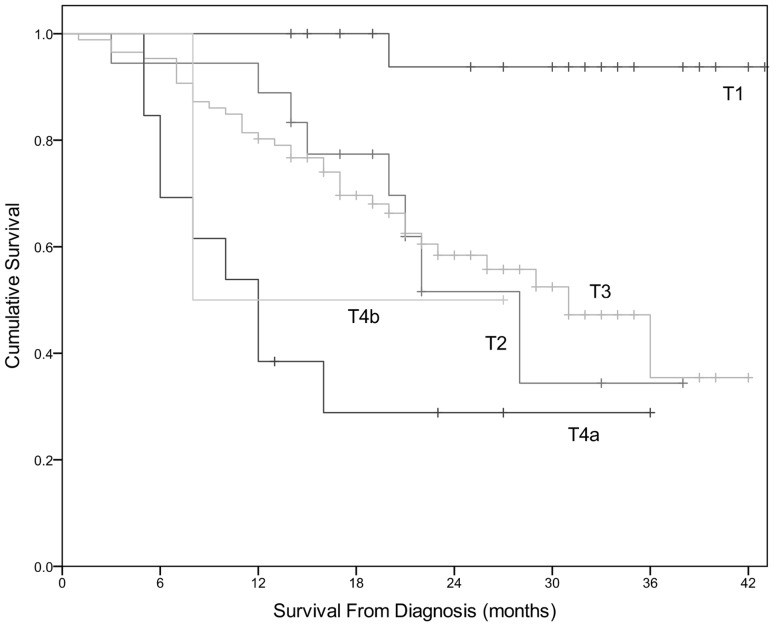
Significant difference in cumulative survival by EUS T-stage in Validation cohort (*χ*^2^ 21.031, *df* 4, *p* < 0.001). Patients with more advanced EUS T-stage have worse OS

EUS T-stage (*χ*^2^ 21.031, *df* 4, *p* < 0.001) (Fig. 1) and EUS N0 versus N+ (*χ*^2^ 4.300, *df* 1, *p* = 0.038) were significantly associated with OS. EUS N-stage did not show a statistically significant association with OS (*χ*^2^ 5.699, *df* 3, *p* = 0.127). Table 2 shows mean survival data for patients classified by EUS T-stage, N-stage, and EUS N0 versus N+.

**Table 2:** Survival data of patients in validation cohort derived from univariate analysis

EUS variable	Mean OS (months)	95% confidence interval	
		Lower	Upper
T-stage			
T1	41.563	38.834	44.291
T2	25.830	20.062	31.598
T3	27.908	24.484	31.332
T4a	16.846	9.842	23.851
T4b	17.500	4.334	30.666
N-stage			
N0	31.853	28.735	34.971
N1	25.625	21.246	30.004
N2	16.857	11.873	21.841
N3	17.000	17.000	17.000
N+	24.924	20.966	28.882

### Multivariate analysis in validation cohort

Again, two Cox regression models were produced in the validation cohort. EUS T-stage and EUS N-stage were entered in model 1. EUS T-stage was significantly and independently associated with OS (HR 11.656–30.114, 95% CI 0.994–243.079, *p* = 0.011). (Table 3) The EPV ratio was 27.0.

**Table 3:** Results of model 1 multi-variate analysis including EUS T-stage and EUS N-stage in validation cohort

Variable	*p* value	Hazard ratio	*df*	95% confidence interval	
				Lower	Upper
EUS T-stage	0.011		4		
T2	0.018	12.482	1	1.528	101.957
T3	0.016	11.656	1	1.570	86.548
T4a	0.001	30.114	1	3.731	243.079
T4b	0.050	16.270	1	0.994	266.273
EUS N-stage	0.553		3		
N1	0.560	1.192	1	0.660	2.154
N2	0.353	1.653	1	0.572	4.772
N3	0.260	3.176	1	0.425	23.716

EUS T-stage and EUS N0 versus N+ were entered in model 2. Again, EUS T-stage was significantly and independently associated with OS (HR 11.714–29.631, 95% CI 1.067–238.959, *p* = 0.012). (Table 4) The EPV ratio was 27.0.

**Table 4:** Results of model 2 multi-variate analysis including EUS T-stage and EUS N0 versus N+ in validation cohort

Variable	*p* value	Hazard ratio	*df*	95% confidence interval	
				Lower	Upper
EUS T-stage	0.012		4		
T2	0.020	11.977	1	1.469	97.620
T3	0.016	11.714	1	1.579	86.902
T4a	0.001	29.631	1	3.674	238.959
T4b	0.045	17.243	1	1.067	278.714
EUS N0 vs N+	0.359	1.292	1	0.747	2.235

There was no statistically significant difference in OS between the development (mean OS 33.0 months, 95% CI 30.060–36.076) and validation cohorts (mean OS 29.8 months, 95% CI 27.120–32.513) (*χ*^2^ 1.979, *df* 1, *p* = 0.159).

### Effect of including site 1 patients in development cohort

A post hoc analysis was performed to determine the effect of including patients scanned in site 1 on the development cohort prognostic models. To perform this post hoc analysis, site 1 patients were excluded from the development cohort in an attempt to control comparison with the validation cohort. Seventy patients were originally scanned at site 2. Of these, 53 patients (75.7%) were staged EUS N0; 11 (15.7%) were EUS N1; 3 (4.3%) were N2, and 3 (4.3%) were N3. Both EUS N-stage (HR 2.365–32.757, 95% CI 0.476–223.922, *p* = 0.005) and EUS N0 versus N+ (HR 3.783, 1.141–12.539, *p* = 0.030) were independent predictors of OS, in keeping with findings from the original study. Therefore, inclusion of site 1 patients had little effect on the prognostic models of the development cohort. Confidence intervals are wide, likely due to the small numbers in N2 and N3 groups.

### Comparison of patient eligibility for development and validation cohorts

A comparison of the proportion of patients who were staged N0 and considered for inclusion during both study periods was made. Post hoc review revealed that 117 of 207 patients (56.5%) from site 2 were staged N0 on PET/CT during the study recruitment period of the development cohort and 139 of 317 (43.8%) from site 2 were staged N0 during the study recruitment period of the validation cohort. This difference was statistically significant (*χ*^2^ 8.049, *df* 1, *p* = 0.005). A significantly higher proportion of patients from site 2 were staged N0 in the development cohort, suggesting that different proportions of patients were eligible for inclusion, potentially affecting the results of the prognostic models.

### Prognostic significance of pathological lymph nodes

**Fig. 2:**
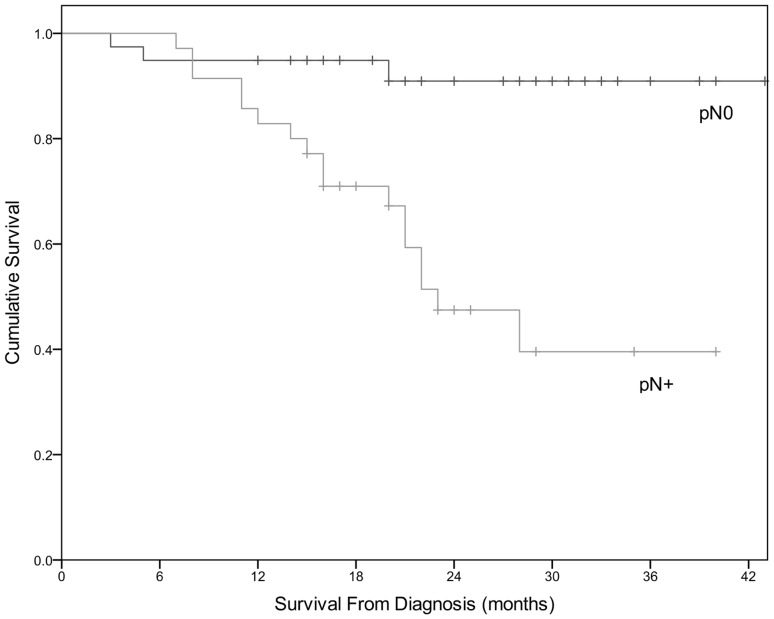
Significant difference in cumulative survival depending on the presence of pathological lymph nodes (*χ*^2^ 13.315, *df* 1, *p* < 0.001). Patients with positive pathological lymph nodes have worse OS

In total, 74 patients from the validation cohort underwent surgical resection. Thirty-nine patients (52.7%) were classified pN0 and 35 patients (47.3%) were classified pN+. There was a significant difference in OS between pN0 and pN+ groups (*χ*^2^ 13.315, *df* 1, *p* < 0.001). (Fig. 2) Mean OS for the pN0 group was 40.091 months (95% CI 36.931–43.251) and 26.538 (95% CI 22.123–30.953) for the pN+ group.

## Discussion

This validation study failed to replicate the results of the developed prognostic model. In the validation cohort, EUS N-stage and EUS N0 versus N+ did not show prognostic significance in multi-variate analysis, although EUS N0 versus N+ was statistically significant in univariate analysis (*p* = 0.038). Relatively small numbers of patients staged EUS N2 and N3 were included in the development cohort (*n* = 9 and *n* = 7, respectively) which could result in disproportionate statistical significance and failure to validate this variable. Importantly, this study showed that EUS T-stage is significantly and independently associated with OS, which supports other studies [14–16]. The study adds evidence to the importance of EUS in the multi-modality approach to oesophageal cancer staging.

When patients scanned in site 1 were removed from the development cohort, EUS N-stage and EUS N0 versus N+ remained independent predictors of OS. This finding suggests that the site 1 PET/CT scanner had little effect on the developed prognostic model. One reason for the inability to validate the model could be the statistically significant difference in proportions of patients staged N0 during both study periods, resulting in fewer patients from site 2 eligible for inclusion in the validation study recruitment period (those staged N0 on PET/CT).

An important issue in validation studies is the extent to which the included cohorts differ. This can result in validation failure unless appropriately adjusted for [17]. Reporting trends over time have not been assessed in this study, but reporting and context bias have been shown to influence radiologists image interpretation [18]. It is possible that a priori knowledge of key findings from the developed prognostic model may increase the likelihood of equivocal lymph nodes on PET/CT being called positive. This awareness of having been studied with consequent impact on behaviour, the so-called ‘Hawthorne effect’, could have contributed to the change in results [19]. However, this is merely a hypothesis and cannot be concluded from these data. In addition, operators were not fully blinded to the results of the PET/CT, potentially influencing the interpretation of EUS N-stage. The final report of the PET/CT is routinely checked prior to EUS to ensure that no distant metastases are detected, preventing inappropriate EUS examination.

Prior to the opening of site 2 in 2010, patients were scanned at site 1 using a Philips 16 section Gemini GXL dedicated PET/CT system (Philips Medical Systems, Cleveland, USA). The two sites used different scanners and protocols, and patients were scanned at different activity uptake times. Patients were scanned at 60 min of uptake time in site 1 and after 90 min in site 2. Longer uptake times lead to higher tumour to background avidity and can therefore increase the conspicuity of lymph node metastases. Secondly, the scanner in site 2 had a time-of-flight (TOF) algorithm but the site 1 scanner did not. TOF reconstructions improve signal-to-noise ratio, detection and anatomical localisation of lymph node metastases by allowing more precise measurement of the time difference between detections [20]. Finally, images were acquired for 4 min per bed position in site 1, whereas the acquisition was 3 min per bed position in site 2. Some improvement in image quality may be expected in site 1 with longer acquisition times, provided the patient remained still. However, the results of this validation study assume that longer acquisition did not affect the models. Standardised PET acquisition protocols have been discussed in the literature [21], but these findings suggest the differences may be less influential than previously thought, adding generalisability to the results.

An additional hypothesis for the failure of validation is the accuracy of the staging investigations. Results from our institution have shown suboptimal diagnostic accuracy in the general oesophageal cancer population [22]. Overall accuracy of differentiating negative and positive nodal disease on CT, EUS, and PET/CT was 54.5, 55.4, and 57.1%, respectively. Sensitivity and specificity were 39.7 and 77.3% with CT, 42.6 and 75.0% with EUS and 35.3 and 90.9% with PET/CT. These results were largely attributable to the detection of micro-metastases in 44% of lymph nodes (defined as ≤ 2 mm) evaluated pathologically. The suboptimal accuracy could affect results of the developed prognostic model, obtaining a significant difference in survival between EUS N-stage categories by chance alone.

The additional sub-group analysis conducted in this study confirms the presence of lymph node metastases as a major prognostic indicator [2]. There was a highly significant difference in OS between pN0 and pN+ groups in patients staged N0 on PET/CT. This finding highlights the importance of accurate pre-treatment lymph node staging in oesophageal cancer.

Model validation is not commonly performed in prognostic research. Approximately 34% of models are validated, with 11% undergoing external validation [23]. The importance of rigorous study design and statistical analysis cannot be understated, and further research is required to improve prognostic research methodology [24, 25]. In addition, it is important to document and publish the findings of all prognostic research, including ‘non-significant’ findings, since publication bias is prevalent [26].

This validation study is limited by some factors. Both development and validation cohorts represent a relatively heterogeneous cohort of patients; however, this is reflective of the demographics of oesophageal cancer patients in general. Dissemination of lymph node metastases is dependent on T-stage and to a lesser extent, histological cell type of the primary tumour [27, 28]. Stratification of these factors was not performed, but most patients presented with locally advanced T3 or T4 tumours. The EUS operators may have been influenced by results of the PET/CT reports, resulting in inadvertent changes in lymph node assessment over time. In addition, the inclusion of patients from site 1 could not be replicated in the validation cohort because our patients no longer attend this site for PET/CT.

Despite the limitations, this validation study has several strengths. All patients were managed by an experienced Upper gastrointestinal cancer multi-disciplinary team covering a population of approximately 1.5 million. The site 2 PET/CT scanner and acquisition protocol was unchanged in both development and validation cohorts. The study was adequately powered according to a recommended EPV ratio. In addition, a pathologist reviewed the resected lymph nodes to evaluate and confirm the presence of pathological lymph node metastases.

## Conclusion

Validation studies are important in prognostic research [29]. Lymph node metastases have a significant detrimental effect on OS. This validation study did not replicate the results of the developed prognostic model but the continued benefit of EUS in patients staged N0 with PET/CT was demonstrated. EUS remains a valuable component of a multi-modality approach to oesophageal cancer staging.
